# Supplementary material for: Splice-Junction-Based Mapping of Alternative Isoforms in the Human Proteome
Source: Cell Rep. Author manuscript; Available in PMC 2020 Jan 15. (PMC6961840; doi:10.1016/j.celrep.2019.11.026)
Supplement: 2 [file NIHMS1546469-supplement-2.zip › DF1/PXD006675/CardiacFibroblast/CardiacFibroblast_13_NEXN_RAEQVNEDEENQDTAK.pdf]

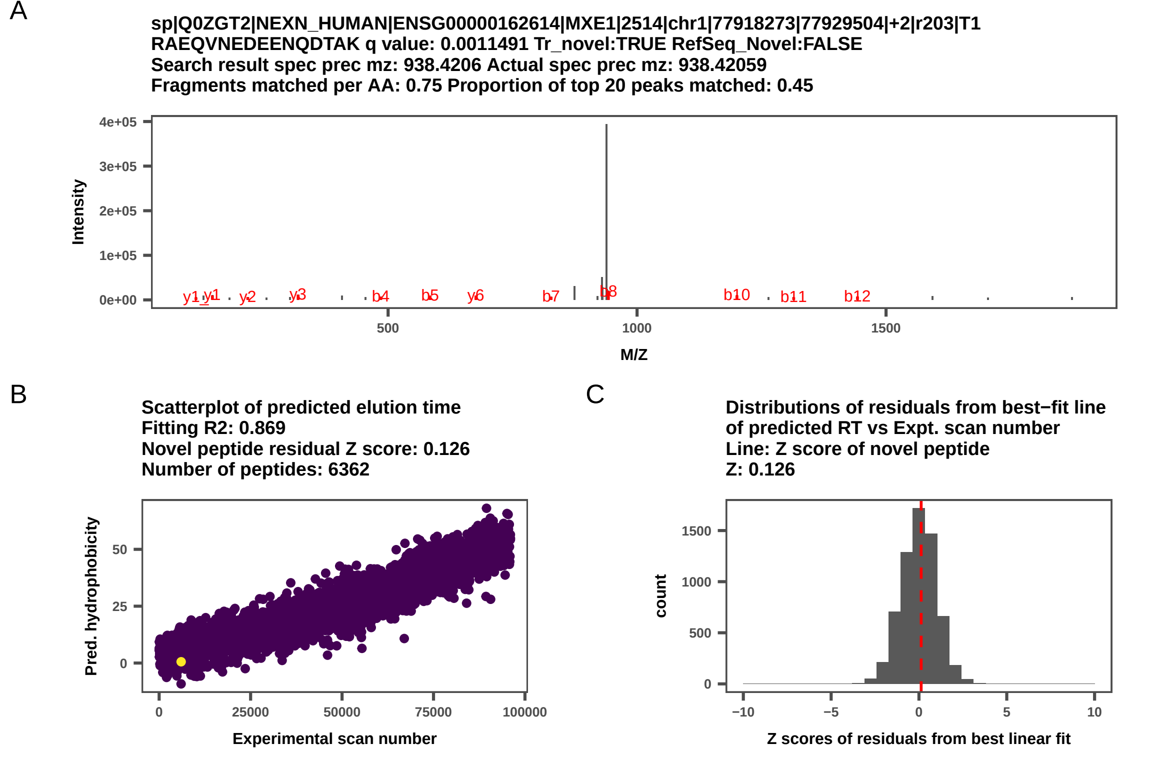
A
sp|Q0ZGT2|NEXN_HUMAN|ENSG00000162614|MXE1|2514|chr1|77918273|77929504|+2|r203|T1
RAEQVNEDEENQDTAK q value: 0.0011491 Tr_novel:TRUE RefSeq_Novel:FALSE
Search result spec prec mz: 938.4206 Actual spec prec mz: 938.42059
Fragments matched per AA: 0.75 Proportion of top 20 peaks matched: 0.45
Intensity
4e+05
3e+05
2e+05
1e+05
0e+00
y1_1
y1
y2
y3
b4
b5
y6
b7
b8
b10
b11
b12
500
1000
1500
M/Z
B
Scatterplot of predicted elution time
Fitting R2: 0.869
Novel peptide residual Z score: 0.126
Number of peptides: 6362
Pred. hydrophobicity
50
25
0
0
25000
50000
75000
100000
Experimental scan number
C
Distributions of residuals from best−fit line
of predicted RT vs Expt. scan number
Line: Z score of novel peptide
Z: 0.126
count
1500
1000
500
0
−10
−5
0
5
10
Z scores of residuals from best linear fit
